# Objectivity, honesty, and integrity: How American scientists talked about their virtues, 1945–2000

**DOI:** 10.1177/00732753231206773

**Published:** 2024-01-08

**Authors:** Kim M. Hajek, Herman Paul, Sjang ten Hagen

**Affiliations:** Leiden University, Netherlands; Leiden University, Netherlands; Utrecht University, Netherlands

**Keywords:** virtue, epistemic virtues, scientific attitude, physics, psychology, history, integrity

## Abstract

What kind of people make good scientists? What personal qualities do scholars say their peers should exhibit? And how do they express these expectations? This article explores these issues by mapping the kinds of virtues discussed by American scientists between 1945 and 2000. Our wide-ranging comparative analysis maps scientific *virtue talk* across three distinct disciplines – physics, psychology, and history – and across sources that typify those disciplines’ scientific ethos – introductory textbooks, book reviews, and codes of ethics. We find that, when inducting students into a discipline, evaluating peers, or codifying their professional standards, postwar American scientists routinely named virtues like carefulness, objectivity, and honesty. They applied such virtues not only directly to scholars’ characters, minds, and attitudes (thereby equating virtues with *personal qualities*), but also to their methods, modes of reasoning, and working habits (in the form of what we call *virtue-qualifiers*). Strikingly, we find that physicists, psychologists, and historians drew upon largely similar repertoires of virtue. For all of them, scientific work required carefulness, thoroughness, and accuracy. Not all virtues, however, were equally important in all disciplines (notably objectivity), nor did each ethos-forming genre place equal emphasis on the directly personal nature of such virtues. All in all, our research establishes an extended framework for understanding the ways virtues remained present in postwar American scientific discourse writ large.

## Introduction

What kind of person makes a good scientist? Does scholarship demand certain personal qualities of its practitioners? Prior to the twentieth century, these were routine questions, recognizable to every scholar. As Steven Shapin and others have demonstrated, early modern natural philosophers frequently talked about virtues relevant to the life of the mind.^1^ Not only were they convinced that scholarship required character traits like impartiality, humility, and perseverance, but they also believed that scholarly activity had an ennobling effect on the character of its practitioners. Learning, in other words, had a doubled relationship to virtue: it both demanded and fostered virtuous ways of being and acting.^2^ Newer research on scholarly virtues and vices has shown that the first belief, that good scholarship requires virtue, remained of vital importance throughout the eighteenth, nineteenth, and early twentieth centuries.^3^ While the second belief – science making better people – gradually disappeared into the background, the importance of certain qualities for doing science continued to receive vigorous attention, especially as fields of learning established themselves as academic disciplines.^4^ Professionalization prompted reflection on the differences between scientists and amateurs as well as on the possibilities and limitations of scientific education (to what extent could scholarly virtues be taught?).^5^ Meanwhile, scholarly virtues (“objectivity”) and vices (“dogmatism”) played key roles in post-Darwinian controversies and other debates at the intersection of science, politics, and religion.^6^ For the United States, it has been shown that virtues were a privileged idiom for evaluating scholarly accomplishments until at least the 1930s or 1940s.^7^ We can ask, however, to what extent this changed later in the century.

Where existing scholarship touches on this question, it does so by identifying two interrelated developments.^8^ First, historians such as Andrew Jewett have observed that scholars in the 1920s and 1930s United States replaced the notion of a “scientific spirit,” defined in terms of personal virtues, with that of a “scientific attitude.”^9^ Arguably, this does not tell us much about the vicissitudes of virtue terms like impartiality, honesty, and accuracy. In earlier centuries, virtue terms had also been grouped under different labels, such as “duties,” “tasks,” and “responsibilities.” Secondly, it has been argued that the declining popularity of “virtue” as an ethical umbrella term, combined with “the disappearance of the individual” and the instrumentalization of science for political and military purposes, caused the ties between science and virtue to wither.^10^ On the one hand, this would have meant that “by the middle of the twentieth century, explicit talk about virtue had largely disappeared from discussions of science,” as Thomas Stapleford and Emanuele Ratti propose.^11^ Where this refers to scientists’ use of virtue terms like honesty and accuracy, however, the argument still needs to be tested empirically: scholars have not yet provided substantial evidence for or against (a gap that this article aims to fill). On the other hand, the literature is replete with master narratives of modernization, bureaucratization, and secularization that tend to associate virtue with the prehistory of modern science – a time when learning was still largely an individual, religiously inspired pursuit – more than with twentieth-century science. What such narratives fail to appreciate, in the view of Steven Shapin, is that “people and their virtues” continued to matter “to the making and the authority of late modern bodies of technical knowledge,” even or precisely in the most advanced branches of American technoscience.^12^ In sum, the theme of scholarly virtues in the twentieth century has been touched upon, but not nearly with the degree of empirical rigor that scholarship on earlier periods has attained. This paper begins to fill this gap by mapping routine discourse about virtues across three scholarly genres in disciplines spanning the full range from the humanities to the physical sciences.

For a historical study of scholarly virtues in postwar science – preferably in the United States, given the global prominence of American science in the postwar period – two things seem crucial: a clear definition of terms and a research design that encompasses more than isolated case studies.^13^ Regarding definitions, we use the term “virtues” as synonymous with personal qualities, character traits, or habits of mind that scholars invoked when discussing the personal demands that science made on its practitioners. “Scholarly virtues,” then, are specifically qualities, traits, or habits that scholars *qua scholars* were expected to possess, cultivate, or display.^14^ This is not a definition likely to be found among virtue ethicists or virtue epistemologists, who usually work with more narrowly construed accounts of moral and intellectual virtue.^15^ Our definition does, however, align closely with historical scholarship on earlier periods, which has recognized the fuzzy boundaries between the epistemic and the ethical in scholars’ talk of virtue as well as scholars’ constant negotiation, in terms of virtues and vices, of political and religious demands made on research and teaching.^16^ “Scholarly virtues” is consequently an analytical category rather than an actor’s term: it denotes a historically evolving set of personal qualities (accuracy, honesty, impartiality, loyalty, thoroughness, objectivity, open-mindedness, and so forth) that scholars invoked in specifying the characteristics of a good scholar.^17^ What we will map, then, is not the changing popularity of the umbrella term “virtue,” but the configurations of specific virtue terms (objectivity, honesty, integrity, etc.) in postwar scientific discourse.

We have two reasons for focusing on scientific discourse about virtues – *virtue talk*, as we call it – rather than on personal qualities that scientists actually possessed or displayed. First, while there is no lack of sources referring, for instance, to Albert Einstein’s curiosity or Thomas Huxley’s dogmatism, such attributions of virtue and vice usually tell us as much about the writers as about the individuals discussed. Similarly, although biographies and obituaries are notoriously unreliable when it comes to the virtues they ascribe to past scientists, such genres do showcase how virtues and vices served as a vocabulary for articulating ideals and expectations regarding scholars’ conduct and working manners.^18^ Secondly, as Lorraine Daston and Peter Galison remind us, such idioms have histories: they evolve over time.^19^ New virtues (open-mindedness, fair-mindedness) might be added, while others (piety, humility) could lose the prominence they once had.^20^ There are multiple historical examples of scholars engaging critically with such shifting valorizations.^21^ Indeed, controversies about the relative weight of individual virtues have historically been the rule rather than the exception, if only because virtues like patriotism and loyalty (to the nation, the church, or the cause of democracy) stood in tension with more ascetic virtues like impartiality and objectivity.^22^ Virtue talk, therefore, must not be mistaken for a coherent system of moral standards: it served as an idiom for articulating a whole range of sometimes irreconcilable expectations.

The second crucial point that marks our study is its ambitious scope. Focusing on American science in the second half of the twentieth century, we investigate discourse about virtue across disciplines spanning the arc from the natural sciences to the humanities. Rather than studying an isolated context or virtue, we provide the first (to our knowledge) bird’s-eye view of routine discourse about virtue across widely distinct disciplines and across a fifty-five-year period. With this extensive historical map, we gain a sense of the whole constellation of virtues that regularly mattered in the postwar American sciences, social sciences, and humanities. Where previous scholarship on twentieth-century virtues has mostly focused on single virtues (open-mindedness, objectivity), single scientists (Merle Tuve, Edwin Boring), or single disciplines (American historiography), our investigation provides a previously lacking framework for connecting and situating such individual, in-depth studies.^23^ It should be noted that we will often use the term “science” or “scholarship” as shorthand for “sciences, social sciences, and humanities,” and similarly refer both to “scientists” and “scholars.”^24^

Since our map aims to inform and orient future studies of individual virtues in discourse and practice, our approach in this article is deliberately broad and multidisciplinary in scope, exploratory rather than exhaustive in nature. The article proposes to encompass the full repertoire of virtue talk – which virtues were most regularly invoked, and how this varied between disciplines and across time – by examining places where the ethos of science was configured and transmitted as part of routine scholarly activity. Specifically, we focus on three genres in which scholars reflected on their disciplinary ethos or scientific norms: introductory textbooks of the kind read by first-year students, book reviews in scholarly journals, and ethics codes issued by major professional/scholarly societies.^25^ We study these genres in three academic disciplines: a prominent postwar social science – psychology – and two fields more frequently the subject of comparative histories of (epistemic) virtue – physics and history.^26^ That psychologists often aspired to the status of the natural sciences, while American historians were variously housed in social science or humanities faculties, implies that our study also implicitly makes connections across typical scholarly groupings.

In examining the dynamics of virtue talk across these disciplines, we notice that in the period under review (1945–2000), virtue terms were no longer solely attributed to individual persons, as had been common in earlier centuries, but were also associated with ways of working, reasoning, or writing. To mark this difference, we speak, on one hand, about *personal qualities* if virtues – like carefulness – were attached or ascribed directly to individuals. On the other hand, if carefulness was attributed to an activity (measuring temperature) or to scientific output (a monograph), we speak about *virtue-qualifiers*. The claim that an experiment performed by Henry Rowland in 1879 was “a model of careful experimentation” differs crucially from the statement that Rowland himself was a model of carefulness.^27^ In the former statement, “careful” appears less as a distinct character trait than as a method or working habit, while in the latter, carefulness becomes a habit of mind solidly ingrained in the person of Rowland. The first “careful,” in our definition, is thus a virtue-qualifier, part of an “adjectival and adverbial arsenal” of virtue terms that are used, not to refer to an individual’s character, personality, or attitude,^28^ but to describe how scientists conduct their studies – how they perform an experiment, analyze a source, or reason out a conclusion.^29^ The difference is subtle but important: “carefulness” as a noun has a characterological force that the adjective “careful” lacks. Moreover, whereas the noun draws attention to itself, the adjective qualifies something else. To distinguish between personal qualities and virtue-qualifiers notably matters because it can help unpack the often sticky question of whether virtues are applied differently between the scholar and the scholarship.^30^

We attend to the prevalence and function of both kinds of virtue talk in three successive sections of this paper, with an eye to personal qualities that are presented as necessary or desirable for scientific performance and to virtue-qualifiers linked to scientific activities. Each section focuses on a particular genre – introductory textbooks, scholarly book reviews, codes of ethics – and compares virtue talk across the disciplines of physics, history, and psychology. While all three genres are sites for communicating and fashioning disciplinary and scientific identities, each has its particular audience and aims. For coherence, we focus on texts linked to American professional scholarly societies and their major review journals: the American Psychological Association (APA) and *Contemporary Psychology* (*CP*; later *PsycCRITIQUES*); the American Historical Association (AHA) and *The American Historical Review* (*AHR*); the American Physical Society (APS) and *Physics Today* (*PT*). For each discipline, we also purposively sampled a selection of U.S. introductory textbooks (see Table A1). Where possible, we have selected our corpus to span the decades from the end of the Second World War to the end of the century (i.e. about 1945–2000).^31^ Thus, we have studied all codes of ethics issued by the three professional societies (see Table A2), although only the APA promulgated ethical standards from the 1950s. Next, we read book reviews from the first issue per year for each of *AHR* and *CP*, and the first five issues per year of *PT*, for an arbitrary year per decade.^32^ Our analysis of U.S. introductory textbooks, finally, draws on a corpus of twenty-one widely used textbooks in physics, psychology, and history, with consideration of multiple editions where available, for a total of thirty-eight texts.^33^

From a close reading of relevant passages, we collate the most frequently invoked virtues and compare them across fields and time. Given that our aim is to map the ways in which virtues were called out in twentieth-century American science, we are not concerned with how (or whether) any of these virtues were enacted in scientific practice. Our discursive map should, however, provide a solid framework from which to address such important questions in future studies. Nor do we make any claim to chart comprehensively or quantitatively how any named virtue occurred over time or genre. In our penultimate section, we nonetheless offer some brief reflections on the changing meanings and importance attached to “objectivity” and “integrity.” Overall, we find that psychologists, historians, and physicists regularly mobilized personal qualities as they inducted students into a discipline, evaluated their peers, and encoded a certain image of their discipline in ethical standards. Our sources, however, reveal a preference for using virtue-qualifiers, especially in book reviews and textbooks, which in turn suggests further important twists to be explored in a longue durée history of scholarly virtues.^34^ Our distinction between personal qualities and virtue-qualifiers notably seems promising in this regard.

## Introductory textbooks

Student textbooks are a good place to start. As a genre aimed at initiating first-year students “into the well-established views and practices of specific scientific communities,” textbooks typically included some discussion of what research in the discipline looked like and how (aspiring) researchers were expected to behave.^35^ As Ernest Hilgard’s *Introduction to Psychology* (1953) put it straightforwardly: “The first question a prospective psychologist must ask himself is this: Am I suited by abilities and interests to become a psychologist?”^36^ In a different idiom, but with similar questions in mind, other authors dwelt on the “qualities,” “personal traits,” “scientific temper,” “scientific attitude,” “habits of mind,” or “skills” required from researchers.^37^

Evidently, therefore, reference to individual qualities remained relevant, as can be seen through a comparison of successive editions (1953, 1962, 1971, 1983, 1996) of Hilgard’s psychology textbook.^38^ Already in the first edition, readers were told that psychology’s scientific ambitions did not allow for “partisanship,” “loyalty to a position held by one or another prominent teacher,” or “doctrinaire” commitment to a favorite theory.^39^ Subsequent editions repeated these warnings, meanwhile adding “stubborn prejudice” and “dogmatism” to the list of vices.^40^ At the same time, they encouraged aspiring psychologists to develop virtuous habits. For instance, the 1962 edition depicted “the objective psychologist” as someone with an “open mind,” “widely modest about what has been achieved so far,” and at times even engaging in “creative thinking.”^41^ Later editions emphasized the importance of “caution” and “careful observation,” while granting the classic virtue of “objectivity” a central place in the moral economy of science: “Objectivity lies at the center of the scientific attitude.”^42^ As if this did not sufficiently illustrate what demands psychological research placed on scientists’ virtues, the 1996 edition made the point explicit by speaking in the first person plural about “our” unvirtuous habits: “We are biased to see other people’s actions as caused by their internal traits . . . This bias often leads us into error.”^43^ Clearly, personal qualities were a matter of concern in all editions (in 1996 perhaps even more than in 1983, in response to a growing interest in “scientific integrity” – a theme to which we return in later sections).

This interest in the scientific self and its defining traits was not unique to the field of psychology: it appeared with equal intensity in history textbooks, and somewhat less prominently in physics textbooks. Most notably, some virtues were advocated in all three disciplines. Almost every title in our sample presented “carefulness” as an indispensable attribute of responsible researchers. Francis Sears and Mark Zemansky’s *University Physics* (1955, 1976, 1987), for instance, abounds with phrases like “note carefully,” “careful attention must be paid,” “it is essential to distinguish carefully,” and “we must always be careful.”^44^ A similar insistence on carefulness permeates David Halliday and Robert Resnick’s *Physics for Students of Science and Engineering* (1960, later reissued as *Fundamentals of Physics*). Over and over, the book uses the adjective “carefully” to specify how rules must be applied, systems defined, curves studied, frequencies chosen, and temperatures measured.^45^ Historians also agreed on the importance of carefulness. Apart from emphasizing repeatedly the need for “careful, thoroughgoing study,” which in practice largely meant “carefully” sifting primary sources and “carefully” testing the reliability of each and every statement, history textbooks summoned their readers to be careful in drawing conclusions “so as not to outrun the evidence.”^46^ Interestingly, the “careful use and interpretation” of source material was not their only concern: they also recommended “great care and patience . . . in achieving proper spelling and punctuation.”^47^ Even more practically, students could learn how to read secondary literature – quickly and selectively if possible, but slowly and carefully when an author’s arguments were at stake – and how to take notes (“be careful to distinguish between your summary and a direct quotation”).^48^ Carefulness was, in other words, a shared virtue that textbook authors in all three fields wanted their readers to develop.

Carefulness did not, however, occupy an equally important place in what we might call the fields’ constellations of virtues. While nobody questioned that carefulness mattered, the relative weight that textbooks attached to it differed significantly both across and within disciplines. While many psychologists resembled physicists by stressing the importance of “careful research” and “careful observation,”^49^ they distinguished themselves from their colleagues in physics by insisting also on objectivity as a defining mark of “the critical, analytical attitude characteristic of the scientific method.”^50^ The 1958 edition of Ruch’s book, for example, stated that terms must be “defined objectively” and measurements made “as objectively as possible.” “Objective observation” apparently was a sine qua non of psychological research.^51^ Later editions elaborated on the theme, noting the “objective, unbiased attitude” or the “objectivity and impartiality” that psychologists must possess.^52^ Concomitantly, Ruch warned students against vices that could detract from objectivity: “prejudices,” “interpretative bias,” and “premature, incomplete, inaccurate conclusion-drawing.”^53^ Clearly, this emphasis on objectivity, sometimes paired with “scientific rigor,” was an attempt to bolster the scientific status of the field.^54^

Just like psychology textbooks, manuals on historical method valorized both carefulness and objectivity. Their ideal researcher similarly exhibited accuracy and thoroughness, but also (at least to some degree) the virtue of objectivity or impartiality. The importance of accuracy and attention to detail is especially prominent in Gilbert Garraghan’s *Guide to Historical Method* (1946).^55^ Similarly, the 1969 edition of Louis Gottschalk’s *Understanding History* portrayed the “painstaking historian” as someone “with an eye for accuracy,” eager to “set down carefully the story he has extracted from the sources.”^56^ These virtues reflect a philological ethos of the kind that source-oriented historians had been cultivating since the mid-nineteenth century.^57^ At the same time, history textbooks emphasized a need for objectivity. Responding either directly or indirectly to Charles Beard’s dismissal of objectivity as a “noble dream,” most of them added that this quality was as valuable as it was difficult to attain.^58^ Garraghan, for instance, wanted historians to develop “a detached and neutral attitude,” in full awareness that absence of all prejudice was “a psychological impossibility.”^59^ Gottschalk added that objectivity was an “obligation,” even if historians “may rarely succeed” in reaching it.^60^ Another textbook spoke in gradual terms about “maximizing objectivity” and “minimizing subjectivity”: “We cannot eradicate all bias, but we can do much to minimize it.”^61^ Although the consensus about this compromise changed over time, what matters for now is that historians, like psychologists but unlike physicists, considered objectivity just as important a virtue as carefulness or accuracy. Constellations of virtues could take on different forms in different disciplines (as well as in different subregions of a field).

Finally, we return to the question of whether all this virtue talk was about *personal qualities* or *virtue-qualifiers*. Were objectivity, honesty, and thoroughness portrayed as traits of personality that researchers had to cultivate? Insofar as these terms were used in noun form, the answer is largely affirmative. “The spirit of objectivity” was unmistakably a personal quality, as it referred to what Robert Daniels called the capacity of “recognizing and regulating one’s own opinions and biases.”^62^ Similarly, adjectives such as “biased” and “unbiased” could be ascribed to the scientist as individual (as in “unbiased observer” and “an individual scientist may very well be biased”), just as “being sensitive” appeared as a personal character trait.^63^ When Ruch argued that even the most careful research design cannot prevent “a poor interviewer” from biasing the results, he made clear that researchers’ personal qualities were a matter of irreducible importance.^64^ Other adjectival forms, in contrast, were less frequently a matter of a scientist’s personal habits. Occasional mention of “the objective psychologist” or “an objective, unbiased attitude,”^65^ for instance, is outweighed in textbooks by references to “objective observation,” “objective data,” “objective measurement,” “objective methods,” “objective validation criteria,” and “objective knowledge.”^66^ Likewise, the adjective “accurate” was predominantly associated not with researchers but with measurements, experiments, predictions, and laboratory notebooks.^67^ In most cases, therefore, virtue talk in textbooks occurred under the sign of virtue-qualifiers: it described other aspects of science than the person of the researcher, such as methods (as in “precise measurements,” “accurate experiments,” “careful manipulation,” and “carefully controlled research”),^68^ modes of reasoning (as in “prove rigorously” and “defining things precisely”),^69^ and working habits (as in “carefully observing,” “rigorous understanding,” and “study these curves carefully”).^70^

## Book reviews

Virtue-qualifiers similarly abound in book reviews, a genre aimed at informing scholars about “the current scene [in a discipline] by telling its readers what the recent books contain” and evaluating the significance of those books.^71^ Notably, the three journals studied here had the additional aim of fostering “a sense of professional unity and morale” in their respective disciplines by providing a space for researchers from different (sub)specializations to learn about “general trends in fields outside their own.”^72^ Book reviews played a central role in furthering this goal. *Contemporary Psychology* was entirely comprised of reviews, while a typical issue of *The American Historical Review* would feature several hundred book reviews to some four or so research articles.^73^

In his instructions to book reviewers, Edwin G. Boring, the first editor of *CP*, pointed to the importance of evaluating books instead of their authors’ habits. “Criticize the text, the ideas, the logic, the accuracy, not the author,” he wrote. “Let all criticism be *ad verbum*, never *ad hominem*.”^74^ For the most part, reviewers complied with this request, not only in *CP*, but also in *AHR* and *PT*. In pointing to “fair-minded . . . work” or “careful and meticulous explanations,” they applied virtue adjectives either to a book’s contents – its analysis, explanation, or interpretation – or to its author’s mode of reasoning, working habits, or methods (translation, transcription, experimentation, interviewing). That is, virtues appeared not in the form of personal qualities but in the form of virtue-qualifiers.^75^

Most common in our sample of book reviews were derivatives of the virtues of “carefulness” and “thoroughness,” notably “careful” and “thorough.” In *CP* and *PT*, books were lauded for their “careful and thorough . . . assessments” of patients or for being “a careful, thorough statement.”^76^ Conversely, they were criticized for containing “uncritical discussions” of quantum theory presented in an “exaggerated style of writing,” with reviews across the three disciplines enlisting variations of virtue-qualifiers of this kind: “balanced,” “nuanced,” “original,” “critical,” and “fair.”^77^ Historian Stanley G. Payne, reviewing a book on the history of Spanish Catholicism, invoked several of these in just a few lines. He praised the author, Frances Lannon, for a work that was not only “valuable and original” but also “sensitive and carefully nuanced.” Moreover, Payne noted that while “Lannon’s analysis is often acerbic and highly critical, [it is] not lacking in basic fairness and judiciousness.”^78^

Some of these virtue-qualifiers were discipline-specific. For example, while “judicious” was a recurring virtue-qualifier in *AHR*, it was largely absent from the other journals.^79^ Disparities between disciplines were especially pronounced, however, when it came to authors’ personal qualities. Notwithstanding Boring’s warning that “*CP* does not provide space for the discussion of the intelligence or integrity of an author . . . personal aspersions are taboo,” postwar American psychologists sometimes critiqued authors as individuals explicitly and directly.^80^ In history as well as in psychology, but not in physics, there was a systematic emphasis on the “empathy” of authors toward their subjects, colleagues, and readers, as well as on the related virtues of “compassion” and “sympathy.” A biographer of Léon Blum was praised not only for being “a meticulous researcher and a fine craftsman,” but especially for “combin[ing] rigorous fairness with a deep-felt sympathy for his protagonist.”^81^ Decades later, Bernardo Carducci was praised in *CP* by the Stanford prison-experimenter Phillip Zimbardo for approaching the topic of shyness “authoritatively” yet “compassionately,” thereby appealing effectively to his intended audience of “shy people.”^82^ Yet if it was chiefly considered positive to affirm that a historian had displayed “warm sympathies for the men about whom he writes,”^83^ or psychologists had “written with an overwhelmingly therapeutic, pastoral, and compassionate orientation,” there were also book reviewers who insisted that an excess of such personal qualities had a distorting effect on historical and psychological scholarship.^84^ An author could have an “oversympathy for his subject,” as one *AHR* reviewer complained.^85^ Similarly, in the first issue of *CP*, an author’s excessive “adoration [and] compassion” for Sigmund Freud was presented as a vice that carried him “headlong through the most obstinate counter evidence when he [was] in the mood to claim another scrap of territory for the Master.”^86^

Besides “empathy” and its cognates, “objectivity” was a virtue invoked by book reviewers in history and psychology alike. Objectivity was said to be a “laudable” personal quality, associated with “candor,” “wisdom,” and “courage.”^87^ The simultaneous appearance of empathy and objectivity as virtues in book reviews in *AHR* and *CP* suggests that scholars held somewhat inconsistent, or even conflicting, views on the attributes of the ideal historian and the ideal psychologist. According to many historians and psychologists, however, the virtues of objectivity on one hand, and compassion, sympathy, or empathy on the other, were actually compatible when neither was taken to excess. Thus, psychologists judged their colleagues on whether they had managed to balance “their objectivity with the need to be appropriately compassionate,” or to “walk the fine line between being detached scientists and being sympathetic observers.”^88^ Historians sought to achieve a similar balance between an empathic and a detached scholarly attitude, as was the finding of a review in *AHR* of Bonnie Smith’s *The Gender of History*. In the review, Smith’s scholarly attitude toward the work of nineteenth-century women historians was praised for being “respectful . . . but, ultimately, dispassionate.”^89^

Given the tight link that historians and psychologists established between objectivity (or “dispassion”) and “scientific” method, it is remarkable that the virtue of “objectivity” was virtually absent in book reviews in physics. This accords with what we find for introductory textbooks; there seems to have been little need to assert the “scientific” status of physics in the Cold War context.^90^ The reviews published in *PT* occasionally mention other personal qualities, however, including “imagination” and “enthusiasm,” as well as “dogmatism” and “criticism.” In 1967, for instance, T. Teichmann emphasized that readers of *An Introduction to Probability Theory and its Applications* “will be grateful to [author William] Feller for his perseverance and enthusiasm in completing his venture,” while British–American quantum theorist Freeman Dyson criticized the authors of a book on quantum field theory for lacking “a critical and enquiring mind.”^91^

That the naming of personal qualities was very much present in postwar U.S. academic book reviews is further illustrated by the many instances in which book reviewers explicitly evaluated the “minds” of their fellow scholars. Minds could be praised for being “broad and penetrating” and “relentlessly probing [and] sensitive,” or, as in Dyson’s review, criticized for being insufficiently “critical.”^92^ In addition, book reviewers explicitly criticized authors’ attitudes, and diagnosed some psychologists with a “partisan attitude,” or, worse, a “cavalier attitude towards evidence.”^93^ Historians’ attitudes, in turn, were praised for exhibiting maturity and responsibility.^94^

## Codes of ethics

Scholars’ minds were also at issue in professional codes of ethics. Although ethics codes have been criticized as “symbolic resources” that express “power relations” in a profession,^95^ they are also valuable (and understudied) sources insofar as they offer glimpses of the attitudes or comportments that made up a field’s self-image.^96^ This self-image often came with specific expectations of practitioners, as is apparent from the prominence of personal qualities in the three societies’ ethics codes. Thus, it is the only one of our three genres to give an equally important, if not more important, place to personal qualities as to virtue-qualifiers. Almost all the codes in our corpus begin by naming some personal quality – the exception is the first set of ethical standards issued by the APA (in 1953), which notably framed its nearly 200 pages around ethical “problems” and example “incidents.” The APS, for instance, began its guidelines for physicists’ professional conduct with the personal quality of honesty: the first paragraph elaborates on the notion that “honesty must be regarded as the cornerstone of ethics in science.”^97^ This prominence of honesty in the physicists’ ethics code is all the more striking as the APS only promulgated one such document during the twentieth century (in 1991), of comparatively brief length. Other personal qualities also make an appearance in these few paragraphs, notably objectivity, effectiveness, and (professional) integrity, which are held to characterize the physicist engaged in ethical behavior. A situation involving a “conflict of interest” can be recognized, according to the APS, by the absence of these qualities.^98^

Of the four personal qualities that characterized the ethical physicist, it is on integrity that various APA and AHA ethics codes repeatedly insisted. Somewhat surprisingly, given our findings in regard to textbooks and book reviews, objectivity was not central to the way psychologists or historians framed their “core set of shared values.”^99^ Integrity, in contrast, headed principles in the 1974 AHA and 1992 APA ethics codes, merited mention in the preamble to the 1959 APA code, and was emphasized (in bold or italics) in both research and teaching sections of the 1987 and 1999 AHA professional standards.^100^ In other words, integrity was labeled prominently as crucial to the make-up of a good historian, and relatively consistently to that of a good psychologist.

But if cultivating integrity certainly made for good scholarship, can we categorize it as properly a virtue? Always a noun attached to the individual historian, psychologist, or physicist, does it count as a personal quality in the sense of a habitual trait of personality or character? Several APA ethics codes point toward answering in the affirmative. “He maintains integrity with respect to the facts of his science and in his relationships with other psychologists and with the public,” declared the preamble to the 1959 code, while the 1992 revision has psychologists as seeking to “promote integrity in the science, teaching, and practice of psychology,” under the principle titled “integrity.”^101^ Here, integrity appears as a quality that mediates or configures a psychologist’s approach to various aspects of his (not yet her) scholarly activity. Like an Aristotelian virtue, it would seem to induce its possessor to act in the right way. If interpreted in these terms, “integrity” would join the constellation of scholarly virtues alongside the more traditional honesty and objectivity.^102^

A somewhat different interpretation emerges, however, if we read on further in the 1992 APA code or analyze statements by the AHA from 1974 onward about “integrity in research” and “integrity in teaching.” The principle of integrity means that “psychologists are honest, fair, and respectful of others,” according to the 1992 APA ethics code.^103^ For its part, the AHA glossed “integrity in teaching” along similar lines as a matter of “intellectual honesty,” “fairness in judging students’ work,” and “open-mindedness” in discussions with students.^104^ Among other things, “integrity in research” asked of historians “an awareness of one’s own bias and a readiness to follow sound method.”^105^ Such statements construe integrity less as an autonomous personal quality and more as an umbrella virtue that groups together a range of personal qualities (honesty, awareness) and even some virtue-qualifiers (fair judgment).

“Competence” takes on much the same dual characteristics in historians’ and psychologists’ ethics codes. “Competence in a field of historical scholarship includes . . . methodological rigor, capacity for interpretation, originality, thoroughness, and skill in writing,” we read in the 1974 AHA document.^106^ Moreover, we find a principle titled “competence” in every successive revision of the APA ethics code from 1959 to 1992, with “competence” even numbered ahead of “integrity” in the 1992 code.^107^ “The maintenance of high standards of competence is a responsibility shared by all psychologists in the interest of the public and the profession as a whole,” it was affirmed in APA documents between 1959 and 1981.^108^ Here, maintaining competence was particularly linked to the psychologist’s self-awareness of her own “limitations” and “effectiveness.”^109^ If the precise wording differs between revisions of the APA code, the repetition of verbal forms of “recognize” reveals the importance of a quality of (self-)awareness for psychologists, just like an “awareness of bias” was an important component of the quality of integrity for historians.

(Self-)awareness had a larger presence in scholarly ethics codes than as an element of competence or integrity, however, especially for psychologists. This is especially the case if we consider not only direct mention of “awareness” or “being aware,” but also the broader conceptual grouping that includes notions of “recognition,” “sensitivity,” or “sensible regard.” A psychology student should be inculcated with a “sensitivity to the ethics of the psychologist-client relationship,” that the psychologist “shows sensible regard for the social codes and moral expectations of the community in which he works,” and as a teacher, is “aware of the fact that their personal values may affect the selection and presentation of instructional materials.”^110^ To be aware of a plurality of moral expectations or perspectives on some material is also to be open-minded, a psychological characteristic that permeated American culture during the Cold War.^111^

It is thus perhaps unsurprising that this larger sense of (self-)awareness is the principal personal trait to sit under both our umbrella qualities of integrity and competence. Otherwise, the more traditional virtues divide surprisingly cleanly between the two, across ethics codes from both the APA and AHA. Under integrity, we find honesty, fairness, open-mindedness, and promptness, while competence can be read as entailing originality, thoroughness, clarity, accuracy, and care.^112^ Recommendations about using good method to promote both integrity (readiness to follow sound method) and competence (methodological rigor) appear in ethical documents directed to historians, but method is hardly embodied in a researcher.

Yet if integrity and competence can be distinguished quite neatly in terms of the virtues that fall under their respective umbrellas, the corpus of ethics codes does not lead to any clear answer on how precisely to categorize integrity, competence, awareness or, additionally, “responsibility,” also a heading in many APA ethics codes.^113^ Although they sometimes appear as virtues “that must be kindled,”^114^ their ontological status becomes blurred in statements that term integrity an “issu[e] of professional conduct” for historians, declare that maintaining competence is a “responsibility,” or explain the psychologist’s “responsibility” as a matter of “plac[ing] high value on objectivity and integrity.”^115^ This kind of ontological mixing has also been found in several quantitative investigations of “research integrity” or “values talk” in contemporary corpora that include ethics frameworks or codes of conduct among their sources.^116^ If integrity, competence, awareness, and responsibility were important qualities for American historians and psychologists, the precise ontological status of these notions remained suspended.^117^

Alongside these ambiguous yet important personal qualities, we encounter a certain number of virtue-qualifiers in the societies’ ethics codes, as we might expect from their importance in our other scholarly genres. Such virtue-qualifiers are similarly predominantly positive; they refer to virtues, not vices.^118^ For example, “thorough, fair and objective evaluations” are required for peer review in physics to “serve its intended function,”^119^ and both historians and psychologists were enjoined to “present their credentials accurately and honestly in all contexts.”^120^ Where scholars were to present something – materials to students, research to the public, or position descriptions and reports in academic recruitment – the duo of objectivity and accuracy predominate. In 1953, the APA noted that “in presenting potentially disturbing subject matter to students,” discussion should be “objective and full,”^121^ a point whose virtue-qualifiers are echoed in the 1992 code’s requirement to “present psychological information accurately and with a reasonable degree of objectivity [to students].”^122^ As well as describing any open positions “accurately,” historians selecting candidates for recruitment should ensure “fair professional practice.”^123^ Modesty and caution (or care) joined the constellation of virtues that qualify scholarly presentation, such as when the APA codes explained how to make public statements: “Modesty, scientific caution, and due regard for the limits of present knowledge characterize all statements of psychologists who supply information to the public.”^124^ Historians, for their part, had to “be careful not to present [their findings] in a way that foreclose[d] discussion of alternative interpretations.”^125^

Unusually for our corpus, recommendations in the 1981 APA ethics code about making public statements were constructed partly in negative terms, regarding what such statements “do not contain” – to wit, “a false, fraudulent, misleading, deceptive, or unfair statement.”^126^ If this is a rare case of negative virtue-qualifiers (vice-qualifiers?), other instances of vices in the APA or AHA ethics codes tend to be expressed in noun form and to point unequivocally to a certain behavior. Historians’ individual beliefs, urged the AHA in 1974, could not justify “the intentional use of falsification, misrepresentation, or concealment or the abuse of academic and psychic authority to intimidate students.”^127^ Together with “misrepresentation,” and “abuse” (or misuse – of authority or results), common vicious comportments in the codes included “fabrication” (of data), “discrimination” (on grounds of sex, race, etc.), and, especially from around the 1990s, “plagiarism” and “sexual harassment.”

About the same time that sexual harassment and plagiarism made their appearance as named forms of unethical behavior, some ethics codes also took on an explicit regulatory function.^128^ Notably, the 1992 revision of the APA code was divided into general principles and a number of “*enforceable* rules for conduct.”^129^ Since any enforcement process can only judge behavior, not *who* a scholar is as a person, the document accordingly specified that it only applied to “psychologists’ work-related activities,” not their “purely private conduct.”^130^ By implication, the scholarly self of a psychologist would be split off from the private self of that same individual, even as virtue talk remained prominent.

## The virtues, are they a-changin’?

This significant persistence of virtue talk, taken to encompass both personal qualities and virtue-qualifiers, shows that American scientists continued to name particular virtues, even in an age when the word “virtue” itself fell out of use. Before elaborating on our findings in the conclusion, we would like to draw attention to a number of changes that can be observed in the period under discussion. We will mention three: (1) the increased prevalence of virtue-qualifiers, especially in book reviews; (2) the declining importance of “objectivity”; and (3) the rise of “integrity.”

First, there are some differences between our three genres in the balance of personal qualities to virtue-qualifiers over the decades studied. The two occur more or less equally frequently in ethics codes throughout the period under discussion, and, although virtue-qualifiers outweighed personal qualities in textbooks, the balance between them did not change significantly over time. In book reviews, however, we do observe a shift. Toward the end of the twentieth century, book reviewers in all three disciplines invoked personal qualities significantly less often, while the frequency with which they used virtue-qualifiers remained more or less constant. Even if virtues continued to be discussed by book reviewers in history, physics, and psychology alike, they were referred to increasingly indirectly, in the form of virtue-qualifiers. A possible explanation for this shift may be that postwar scientists felt increasingly uncomfortable with directly mentioning the personal qualities of their peers. We find expression of this sentiment not only in *CP* editor Boring’s advice to book reviewers to avoid ad hominem criticism, but also in the APA’s first ethics code (1953), in which book reviewers were enjoined to focus “on the adequacy of the work done rather than on the integrity or the ability of the author.”^131^ In the 1980s, philosopher of science Larry Laudan echoed such calls when urging his fellow critics of creationism to “assess the merits or demerits of creationist theory without . . . speculat[ing] about the unsavoriness of the mental habits of creationists.”^132^

If book reviewers were increasingly reluctant to indicate authors’ personal qualities directly, it is important to add that they continued to regard the use of virtue-qualifiers as unproblematic. Sometimes, book reviewers were even encouraged to use this form of virtue talk. Interestingly, the AHA’s 2005 code of conduct (an amended version of which is still used) recommends those “reviewing books . . . to evaluate the honesty and reliability with which the historian uses primary and secondary source materials,” thereby linking the virtues of honesty and reliability to researchers’ practices rather than their personality.^133^

A second change relates to “objectivity.” As we saw in the sections on introductory textbooks and book reviews, this virtue was fundamental for both historians and psychologists in the 1950s and 1960s; however, it then declined in the 1970s and 1980s. (Recall that physicists had talked about objectivity only rarely even in the 1950s and 1960s.) In place of triumphant phrases like “objective science of psychology,” discussion of objectivity took the form of more cautious warnings against “bias,” which was increasingly presented as something of which psychologists needed to be aware. Whereas Hilgard’s 1962 textbook had instructed the aspiring psychological researcher to “remain objective in his study of the facts as they exist,” implying that objectivity was something that could be attained as a matter of course, later psychology textbooks confessed that an attitude “uncolored by personal feelings and attitudes” was so “extremely difficult” and “almost impossible” to attain that “clinical practitioners cannot be totally objective no matter how hard they try.”^134^

Historians’ attitudes toward objectivity shifted similarly. While some textbook authors tried to keep the “noble dream” of objectivity in place by arguing that objectivity should not be confused with a total absence of bias, or by granting that it could only be realized to a certain degree, others preferred to abandon the quest, embracing alternative virtues like honesty instead.^135^ Textbook authors specifically recommended historians to reveal their biases, thus allowing readers to recognize them, rather than making vain attempts to overcome them.^136^ Along these lines, a 1977 textbook quoted Gaetano Salvemini: “Impartiality is a dream and honesty a duty. We cannot be impartial, but we can be intellectually honest.”^137^ The 1987 AHA code took a similar stance by recommending “awareness of one’s own bias” as part of a historian’s “integrity in scholarship.”^138^ Historians, in other words, were increasingly summoned not to overcome their biases, but to be transparent about them – a subtle but important difference that marked the end of a long tradition of suppressing personal attitudes and preferences.^139^

Thirdly, “integrity of scholarship,” as discussed in the AHA code, points to what some have identified as a new chapter in the moral history of American science: the emergence of integrity as something between a metonym and an umbrella term for responsible conduct. While most existing literature points to the 1990s as a decade witnessing a quick rise in popularity of “integrity,” we saw that this term already featured prominently in the 1959 APA ethics code, while also occupying a privileged place in the AHA standards from 1974 onward.^140^ In the genre of ethics codes, therefore, the use of integrity as a virtue *term* significantly predates the rise of research integrity as a broad *concept* in academic ethics.^141^ As noted above, however, ethics codes are ambiguous on whether integrity should be classed as a (new) virtue in itself or an umbrella quality. We should also add that our findings here refer only to codes of conduct: textbooks and book reviews did not often invoke integrity, either before or after the 1990s. This may reflect a time-lag in the pervasiveness of integrity talk (if integrity were to appear in such sources in the 2000s or 2010s). Alternatively, it may be a matter of the kinds of genres in which scholars discuss integrity (like policy documents, articles about scientific fraud, or editorials). Integrity is, after all, difficult to use in adjectival form, making it an unlikely virtue-qualifier.

## Conclusion

Comparing textbooks, book reviews, and codes of ethics in history, physics, and psychology, we have observed regular use of virtue talk in all three genres, in all disciplines, over the entire postwar period in the twentieth century. Not only were certain personal attributes and forms of behavior indispensable to the pursuit of research, but historians, physicists, and psychologists continued to name qualities like carefulness, fairness, and honesty explicitly when they talked about the business of science. We can thus extend Shapin’s aforementioned thesis that “personal virtue *still matters* to the making and warranting” of late modern science to scientists’ discourse: *talk* about personal virtues also persisted among American scientists between 1945 and 2000.^142^

In addition, our research maps out some ways this discourse on virtue took on different forms. Postwar American scientists applied virtues both to individuals’ personalities and attitudes (what we call *personal qualities*), and also to their methods, modes of reasoning, and working habits (in the form of *virtue-qualifiers*). Throughout the period under discussion, these two forms of virtue talk co-existed, sometimes in entangled ways. The ratio between them, however, differed across genres. If book reviewers and textbook authors commonly made direct reference to personal qualities, their use of virtue-qualifiers was even greater, especially toward the end of the twentieth century. Ethics codes, by contrast, show a more equal balance between personal qualities and virtue-qualifiers. The most notable divergence between the three genres studied is thus a matter of how directly virtues were attached to the person of the scholar as an individual, not of which virtues they promoted. Indeed, this may well constitute a properly generic difference, linked to the textual particularities of ethics codes, as contrasted with book reviews or textbooks. Ethics codes, after all, tended to be considerably shorter than textbooks (one or two pages), while still having to cover the full range of traits or comportments that went into making a good scholar. We can speculate whether this condensed structure brought a corresponding need to name the desired qualities directly, rather than to describe them, as suggested by differences between the lengthy 1953 APA ethics code and its abbreviated successor in 1959.

Our analysis also points to several differences between fields in the prevalence of virtue talk, and in the repertoire of virtues discussed. “Objectivity” is most notable in being invoked frequently by historians and psychologists, yet largely unmentioned by physicists. That said, similarities in virtue talk across the disciplines were as important as the differences. These similarities are most obvious on the level of virtue-qualifiers. We find that historians, psychologists, and physicists mobilized the same core set of virtue-qualifiers to evaluate their peers’ work, introduce their students to their disciplines, or define their professional standards. In particular, they made frequent use of virtue-qualifiers related to “thoroughness,” “carefulness,” and “accuracy” – virtues central to what has been called an ethos of exactitude.^143^

More broadly, we have sketched out a comparative framework encompassing the constellation of virtues regularly named as relevant to doing science in postwar America, a set that is often not internally consistent, but rather comprises ideals that pull in different directions. This will open the way for future well-researched case studies that extend beyond a single author, genre, or practice. How repertoires of virtue might have articulated with dimensions of gender, race, or class merits particular attention, given that historians have already noted that a virtue like “objectivity” carried with it connotations of whiteness and masculinity.^144^ We already know that non-white and non-male students and researchers generally struggled to be recognized as properly scientific in Cold War America, making it highly pertinent to examine further the ways in which virtue talk was employed as a vehicle of marginalization and exclusion in this context. In addition, it seems worth comparing (academic) scientific disciplines to mixed or applied professions, like medicine, which also has a centuries-long tradition of thinking about virtues, and journalism, where notions of virtue left a deep imprint on some of the field’s earliest codes of ethics.^145^

For such future studies, this article offers an additional analytical resource in the novel distinction made between personal qualities and virtue-qualifiers. Indeed, in its absence, existing scholarship sometimes tends to conflate the situation of researchers embodying the quality of carefulness with research characterized by careful measurement and papers containing carefully worded arguments. Something similar happens in studies of “epistemic virtues,” especially if this category is defined so broadly as to bundle virtues like “objectivity” with what Samuel Schindler calls “theoretical virtues,” that is, properties of scientific theories like testability, coherence, and fertility.^146^ Our distinction between personal qualities and virtue-qualifiers could therefore fruitfully be applied to disentangling distinct elements of scholars’ virtue talk – and indeed to understanding scholarly virtues and vices in general – in earlier periods and other parts of the world. This promises to deepen our insight into the longue durée history of scholarly virtue discourse, as well as to enable us to assess what, if anything, was new about the range of virtues talked about by scientists in the postwar United States.

## Footnotes

1. Steven Shapin, *A Social History of Truth: Civility and Science in Seventeenth-Century England* (Chicago, IL: University of Chicago Press, 1994); Peter N. Miller, *Peiresc’s Europe: Learning and Virtue in the Seventeenth Century* (New Haven, CT: Yale University Press, 2000); Kathryn Murphy and Anita Traninger (eds.), *The Emergence of Impartiality* (Leiden, Netherlands: Brill, 2014).

2. Matthew L. Jones, *The Good Life in the Scientific Revolution: Descartes, Pascal, Leibniz, and the Cultivation of Virtue* (Chicago, IL: University of Chicago Press, 2006); Sorana Corneanu, *Regimens of the Mind: Boyle, Locke, and the Early Modern Cultura Animi Tradition* (Chicago, IL: University of Chicago Press, 2012).

3. Chad Wellmon, *Organizing Enlightenment: Information Overload and the Invention of the Modern Research University* (Baltimore, MD: Johns Hopkins University Press, 2015), pp.234–61; Christiaan Engberts, *Scholarly Virtues in Nineteenth-Century Sciences and Humanities: Loyalty and Independence Entangled* (Cham, Switzerland: Palgrave Macmillan, 2022).

4. Though not without making comebacks, as in the “scientific democracy” tradition studied by Andrew Jewett, *Science, Democracy, and the American University: From the Civil War to the Cold War* (Cambridge: Cambridge University Press, 2012).

5. Markus Krajewski, “Genauigkeit: Zur Ausbildung einer epistemischen Tugend im ‘langen 19. Jahrhundert,’” *Berichte zur Wissenschaftsgeschichte* 39 (2016): 211–29; Alexander Stöger, *Epistemische Tugenden im deutschen und britischen Galvanismusdiskurs um 1800* (Paderborn, Germany: Wilhelm Fink, 2021); Kasper Risbjerg Eskildsen, “Virtues of History: Exercises, Seminars, and the Emergence of the German Historical Discipline, 1830–1900,” *History of Universities* 34 (2021): 27–40; Herman Paul, *Historians’ Virtues: From Antiquity to the Twenty-First Century* (Cambridge: Cambridge University Press, 2022), pp.32–43.

6. Herman Paul and Alexander Stoeger, *Dogmatism: On the History of a Scholarly Vice* (London: Bloomsbury, 2024); Camille Creyghton, “Impartiality, Objectivity, and Political Engagement in Nineteenth-Century French Historiography: Monod and the Dreyfus Affair,” *History of Humanities* 3 (2018): 279–302.

7. Sjang L. ten Hagen, “Evaluating Knowledge, Evaluating Character: Book Reviewing by American Historians and Physicists (1900–1940),” *History of Humanities* 7 (2022): 251–77.

8. Virtue ethical approaches to research integrity typically draw on Aristotle and late twentieth-century virtue ethicists like Alasdair MacIntyre, without engaging with the myriad of ways in which scholarly virtues and vices were taught, debated, and sustained in the centuries in between. See, e.g., Robert T. Pennock’s otherwise stimulating book, *An Instinct for Truth: Curiosity and the Moral Character of Science* (Cambridge, MA: MIT Press, 2019).

9. Jewett, *Science, Democracy, and the American University*, pp.10, 24, 113–14 (note 4); Rebecca B. Miller, “Making Scientific Americans: Identifying and Educating Future Scientists and Nonscientists in the Early Twentieth Century” (Ph.D. thesis, Harvard University, 2017), pp.121–49. This shift was connected, among other things, to the rise of “personality” as a descriptive, scientific, non-moralistic alternative to Victorian-sounding notions of virtue and character. Ian A. M. Nicholson, *Inventing Personality: Gordon Allport and the Science of Selfhood* (Washington, DC: American Psychological Association, 2003).

10. Steven Shapin, *The Scientific Life: A Moral History of a Late Modern Vocation* (Chicago, IL: University of Chicago Press, 2008), p.3.

11. Thomas A. Stapleford and Emanuele Ratti, “Using Virtue to Think about Science and Technology,” in Emanuele Ratti and Thomas A. Stapleford (eds.), *Science, Technology, and Virtues: Contemporary Perspectives* (Oxford: Oxford University Press, 2021), pp.1–13, 2.

12. Shapin, *The Scientific Life*, p.1 (note 10).

13. There are also historiographical reasons for focusing on the postwar United States: it allows for comparison between our findings and those of Steven Shapin, most notably his *The Scientific Life* (note 10), and with broader studies like Pelin Kesebir and Selin Kesebir, “The Cultural Salience of Moral Character and Virtue Declined in Twentieth Century America,” *The Journal of Positive Psychology* 7 (2012): 471–80 (which has nothing specific to say about scientific discourse).

14. Engberts, *Scholarly Virtues*, pp.5–6 (note 3); Jeroen van Dongen and Herman Paul, “Introduction: Epistemic Virtues in the Sciences and the Humanities,” in Jeroen van Dongen and Herman Paul (eds.), *Epistemic Virtues in the Sciences and the Humanities* (Cham, Switzerland: Springer, 2017), pp.1–10.

15. E.g., Robert C. Roberts and W. Jay Wood, *Intellectual Virtues: An Essay in Regulative Epistemology* (Oxford: Clarendon, 2007); Jason Baehr, *The Inquiring Mind: On Intellectual Virtue and Virtue Epistemology* (Oxford: Oxford University Press, 2011).

16. Sari Kivistö, *The Vices of Learning: Morality and Knowledge at Early Modern Universities* (Leiden, Netherlands: Brill, 2014); Engberts, *Scholarly Virtues*, pp.139–73 (note 3); Herman Paul, “The Virtues of a Good Historian in Early Imperial Germany: Georg Waitz’s Contested Example,” *Modern Intellectual History* 15 (2018): 681–709.

17. Historical studies of individual virtues and vices include Barbara M. Benedict, *Curiosity: A Cultural History of Early Modern Inquiry* (Chicago, IL: University of Chicago Press, 2001); Neil Kenny, *The Uses of Curiosity in Early Modern France and Germany* (Oxford: Oxford University Press, 2004); Lorraine Daston and Peter Galison, *Objectivity* (New York, NY: Zone Books, 2007); Murphy and Traninger, *Emergence of Impartiality* (note 1); Eric Schliesser (ed.), *Sympathy: A History* (Oxford: Oxford University Press, 2015); Susan Lanzon, *Empathy: A History* (New Haven, CT: Yale University Press, 2018); Markus Krajewski, Antonia von Schöning, and Mario Wimmer (eds.), *Enzyklopädie der Genauigkeit* (Konstanz, Germany: Konstanz University Press, 2021); Gayle Rogers, *Speculation: A Cultural History from Aristotle to AI* (New York, NY: Columbia University Press, 2021); Silvia Negri (ed.), *Representations of Humility and the Humble* (Florence, Italy: Sismel, 2021).

18. Anna Echterhölter, *Schattengefechte: Genealogische Praktiken in Nachrufen auf Naturwissenschaftler (1710–1860)* (Göttingen, Germany: Wallstein, 2012).

19. Daston and Galison, *Objectivity*, p.40 (note 17).

20. On open-mindedness as a scholarly virtue in Cold War America, see Jamie Cohen-Cole, *The Open Mind: Cold War Politics and the Sciences of Human Nature* (Chicago, IL: University of Chicago Press, 2014).

21. Herman Paul, “Virtue Language in Nineteenth-Century Orientalism: A Case Study in Historical Epistemology,” *Modern Intellectual History* 14 (2017): 689–715; Jo Tollebeek, *Men of Character: The Emergence of the Modern Humanities* (Wassenaar, Netherlands: NIAS, 2011).

22. Creyghton, “Impartiality” (note 6); Engberts, *Scholarly Virtues*, pp.175–206 (note 3).

23. See, e.g.: Steven Shapin, “The Rise and Rise of Creativity,” *Aeon*, October 12, 2020, <https://aeon.co/essays/how-did-creativity-become-an-engine-of-economic-growth> (September 18, 2023); Michael Bycroft, “Psychology, Psychologists, and the Creativity Movement: The Lives of Method inside and outside the Cold War,” in Mark Solovey and Hamilton Cravens (eds.), *Cold War Social Science: Knowledge Production, Liberal Democracy, and Human Nature* (New York Palgrave Macmillan, 2012), pp.197–214; Cohen-Cole, *The Open Mind* (note 20); Jessica Wang, “Physics, Emotion, and the Scientific Self: Merle Tuve’s Cold War,” *Historical Studies in the Natural Sciences* 42 (2012): 341–88; Jessica Wang, “‘Broken Symmetry’: Physics, Aesthetics, and Moral Virtue in Nuclear Age America,” in Jeroen van Dongen and Herman Paul (eds.), *Epistemic Virtues in the Sciences and the Humanities* (Cham, Switzerland: Springer, 2017), pp.27–47; Alexandra Rutherford, “Maintaining Masculinity in Mid-Twentieth-Century American Psychology: Edwin Boring, Scientific Eminence, and the `Woman Problem’,” *Osiris* 30 (2015): 250–71; Peter Novick, *That Noble Dream: The “Objectivity Question” and the American Historical Profession* (Cambridge: Cambridge University Press, 1988).

24. Our analysis encompasses a spectrum of disciplines as wide as captured in the German *Wissenschaft* or French *science*.

25. These remain understudied genres in the history of science, notwithstanding Aleksei Pleshkov and Jan Surman, “Book Reviews in the History of Knowledge,” *Studia Historiae Scientiarum* 20 (2021): 629–50. Bibliographical details of textbooks and ethics codes cited can be found in Tables A1 and A2, respectively.

26. E.g.: Ten Hagen, “Evaluating Knowledge” (note 7); Herman Paul, “Weber, Wöhler, and Waitz: Virtue Language in Late Nineteenth-Century Physics, Chemistry, and History,” in Jeroen van Dongen and Herman Paul (eds.), *Epistemic Virtues in the Sciences and the Humanities* (Cham, Switzerland: Springer, 2017), pp.91–107.

27. Robert Resnick and David Halliday, *Physics*, 2 vols., 3rd ed. (New York: John Wiley & Son, 1977), vol. 1, p.483.

28. Like our use of “virtue,” we take the terms “character” and “personality” relatively loosely and interchangeably to denote the set of traits embodied in an individual, rather than attending to how our actors would have understood the conceptual differences between the two. Personality certainly dominated as a term of analysis in the psychology of the era.

29. We owe the notion of “adjectival and adverbial arsenal” to Amanda Anderson’s discussion of characterological language in the modern humanities. Amanda Anderson, *The Way We Argue Now: A Study in the Culture of Theory* (Princeton, NJ: Princeton University Press, 2006), p.134.

30. For instance, these categories would also allow us to interrogate the extent to which a “scientific attitude” was discussed in terms of an individual’s inherent make-up, as opposed to being a “pose” adopted in certain circumstances or activities.

31. Note that some texts in general use early in the 1950s were in fact published in the late 1940s, just as some ethics codes saw important revisions in the early 2000s.

32. The specific years sampled were 1956, 1967, 1978, 1989, and 2000. Out of a total number of 1,224 book reviews examined, we read the following number per journal and per year: *PT*: 210 (45, 63, 30, 36, 36); *CP*: 179 (19, 29, 30, 49, 52); *AHR*: 835 (57, 146, 250, 205, 177).

33. For history, we read as many methodological texts as available, while we compiled our sample of textbooks for physics and psychology from the lists of texts discussed in existing scholarship on the role of textbooks in science, as well as from lists in disciplinary or publishers’ surveys of popular textbooks. In both physics and psychology, the methods and ethos of the relevant discipline usually appear intermixed into an introduction to key ideas. See e.g. Ivan Flis, “Instructional Manuals of Boundary-Work: Psychology Textbooks, Student Subjectivities, and Disciplinary Historiographies,” *Journal of the History of the Behavioral Sciences* 52 (2016): 258–78.

34. An initial attempt at such a longue durée history, focused on historians’ virtues, is made in Paul, *Historians’ Virtues* (note 5).

35. Marga Vicedo (ed.), “Introduction: The Secret Lives of Textbooks,” *Isis* 103 (2012): 83–7, 83. Recently, historians have argued that textbooks play a larger role in knowledge-making. See papers in the focus section of Marga Vicedo, “Textbooks in the Sciences,” *Isis* 103 (2012): 83–138.

36. Ernest R. Hilgard, *Introduction to Psychology*, 1st ed. (New York: Harcourt, Brace, and Company, 1953), p.586. On American psychology textbooks, see Flis, “Instructional Manuals of Boundary-Work” (note 33).

37. Gilbert J. Garraghan, *A Guide to Historical Method*, Jean Delanglez (ed.) (New York: Fordham University Press, 1946), pp.43, 53, 55; Ernest R. Hilgard, Richard C. Atkinson, and Rita L. Atkinson, *Introduction to Psychology*, 5th ed. (New York: Harcourt, Brace, and World, 1971), p.552; Robert V. Daniels, *Studying History: How and Why*, 3rd ed. (Englewood Cliffs, NJ: Prentice-Hall, 1981), p.11; Conal Furay and Michael J. Salevouris, *The Methods and Skills of History: A Practical Guide* (Arlington Heights, IL: H. Davidson, 1988), p.8; Clifford T. Morgan et al., *Introduction to Psychology*, 7th ed. (New Delhi: McGraw-Hill Education, 1993), p.10.

38. On which see Richard A. Griggs and Sherri L. Jackson, “Forty Years of Introductory Psychology: An Analysis of the First 10 Editions of Hilgard et al.’s Textbook,” *Teaching of Psychology* 23 (1996): 144–50.

39. Hilgard, *Introduction to Psychology*, 1st ed., pp.555, 558, 561 (note 36).

40. Ernest R. Hilgard, *Introduction to Psychology*, 3rd ed. (New York: Harcourt, Brace, and World, 1962), pp.218, 324.

41. Ibid., pp.50, 218, 604, 362.

42. Hilgard et al., *Introduction to Psychology*, 5th ed., pp.400, 15, 552 (note 37). On objectivity as a virtue, see Daston and Galison, *Objectivity* (note 17).

43. Rita L. Atkinson et al., *Introduction to Psychology*, 12th ed. (Orlando, Fl: Harcourt Brace, 1996), p.8. This is a later edition of ‘Hilgard’s’ text.

44. Francis Weston Sears and Mark W. Zemansky, *University Physics*, 2nd ed. (Cambridge, MA: Addison-Wesley, 1955), pp.641, 512; Francis Weston Sears, Mark W. Zemansky, and Hugh D. Young, *University Physics*, 5th ed. (Reading, MA: Addison-Wesley, 1976), p.367; Francis Weston Sears, Mark W. Zemansky, and Hugh D. Young, *University Physics*, 7th ed. (Reading, MA: Addison-Wesley, 1987), p.358.

45. Robert Resnick, David Halliday, and Kenneth S. Krane, *Physics*, 2 vols., 4th ed. (New York: John Wiley & Sons, 1992), vol. 1, pp.239, 257, 319, 422, 495.

46. Garraghan, *A Guide to Historical Method*, pp.139, 151 (note 37); Robert Jones Shafer (ed.), *A Guide to Historical Method*, 2nd ed. (Homewood, IL: Dorsey Press, 1974), p.47.

47. Shafer, *A Guide to Historical Method*, pp.83, 216–17 (note 46).

48. Furay and Salevouris, *The Methods and Skills of History*, pp.55, 61 (note 37); Daniels, *Studying History*, pp.72, 84 (note 37).

49. E.g. Floyd L. Ruch, *Psychology and Life*, 5th ed. (Chicago, IL: Scott, Foresman, 1958), p.165; Clifford T. Morgan and Richard Austin King, *Introduction to Psychology*, 3rd ed. (New York: McGraw-Hill, 1966), p.4; Hilgard et al., *Introduction to Psychology*, 5th ed., p.15 (note 37).

50. Floyd L. Ruch and Philip G. Zimbardo, *Psychology and Life*, 8th ed. (Glenview, IL: Scott, Foresman, 1971), p.33.

51. Ruch, *Psychology and Life*, 5th ed., pp.11, 74, 600 (note 49). “Objectivity” even appeared in the book’s glossary, as “the degree to which two or more persons can score a subject’s responses and get the same results” (p.600).

52. Floyd L. Ruch, *Psychology and Life*, 7th ed. (Chicago, IL: Scott, Foresman, 1967), p.16; Ruch and Zimbardo, *Psychology and Life*, 8th ed., pp.14, 51 (note 50).

53. Ibid., pp.15, 28, 33.

54. Ruch, *Psychology and Life*, 5th ed., p.22 (note 49). On the scientific ambitions of American psychology in this period, see James H. Capshew, *Psychologists on the March: Science, Practice, and Professional Identity in America, 1929–1969* (Cambridge: Cambridge University Press, 1999). David Myers’ textbook of 1989 was exceptional insofar as its ambition to pair “rigorous science” with “a broad human perspective” translated into a catalog of virtues that also included critical thinking, skepticism, humility, and “an attitude of open-minded doubt.” David G. Myers, *Psychology*, 2nd ed. (New York: Worth, 1989), pp.ix, 9.

55. Garraghan, *A Guide to Historical Method*, pp.43, 45, 53 (note 37).

56. Louis Gottschalk, *Understanding History: A Primer of Historical Method*, 2nd ed. (New York: Alfred A. Knopf, 1969), p.17.

57. See Rolf Torstendahl, “Fact, Truth, and Text: The Quest for a Firm Historical Basis for Historical Knowledge around 1900,” *History and Theory* 42 (2003): 305–31; Herman Paul, “Distance and Self-Distanciation: Intellectual Virtue and Historical Method around 1900,” *History and Theory* “Theme Issue” 50 (2011): 104–16.

58. Charles A. Beard, “That Noble Dream,” *AHR* 41 (1935): 74–87. See also Novick, *That Noble Dream* (note 23).

59. Garraghan, *A Guide to Historical Method*, pp.46, 47 (note 37).

60. Gottschalk, *Understanding History*, p.137 (note 56).

61. Robert Jones Shafer (ed.), *A Guide to Historical Method*, rev. ed. (Homewood, IL: Dorsey Press, 1974), pp.173, 169.

62. Daniels, *Studying History*, pp.114, 89 (note 37).

63. Hilgard, *Introduction to Psychology*, 3rd ed., p.7 (note 40); Henry Gleitman, *Psychology*, 2nd ed. (New York: W. W. Norton, 1986), p.292; Morgan et al., *Introduction to Psychology*, 7th ed., pp.10, 5 (note 37).

64. Ruch, *Psychology and Life*, 7th ed., p.588 (note 52).

65. Hilgard, *Introduction to Psychology*, 3rd ed., p.50 (note 40); Ruch, *Psychology and Life*, 7th ed., p.16 (note 52).

66. Ruch, *Psychology and Life*, 5th ed., p.12 (note 49); Hilgard, *Introduction to Psychology*, 3rd ed., p.7 (note 40); Ruch, *Psychology and Life*, 7th ed., p.138 (note 52); Hilgard et al., *Introduction to Psychology*, 5th ed., p.7 (note 37); Gleitman, *Psychology*, 2nd ed., p.612 (note 63); Morgan et al., *Introduction to Psychology*, 7th ed., p. 5 (note 37).

67. Sears and Zemansky, *University Physics*, 2nd ed., pp.1, 273 (note 44); Resnick and Halliday, *Physics*, 2 vols., 3rd ed., vol. 1, pp.338, 339 (note 27); Morgan and King, *Introduction to Psychology*, 3rd ed., p.21 (note 49); Daryl W. Preston and Eric R. Dietz, *The Art of Experimental Physics*, 1st ed. (New York: John Wiley & Sons, 1991), p.5.

68. Sears and Zemansky, *University Physics*, 2nd ed., pp. 2, 273, 379 (note 44); Morgan et al., *Introduction to Psychology*, 7th ed., p.493 (note 37).

69. Robert Resnick and David Halliday, *Physics for Students of Science and Engineering*, 2 vols. 2nd ed. (New York: John Wiley & Son, 1962), p.993; Richard P. Feynman, Robert B. Leighton, and Matthew Sands, *The Feynman Lectures on Physics*, vol. 1. (Redwood City, CA: Addison-Welsey, 1963), p.82.

70. Morgan and King, *Introduction to Psychology*, 3rd ed., p.4 (note 49); Paul A. Tipler, *Physics*, 1st ed. (New York: Worth, 1976), p.4; Resnick, Halliday, and Krane, *Physics*, 2 vols., 4th ed., vol. 1, p.319 (note 45).

71. Edwin G. Boring, “CP Speaks. . .,” *CP* 1 (1956): 13. Historians have found virtue talk to be prominent in American book reviews up to the mid-twentieth century: Ten Hagen, “Evaluating Knowledge” (note 7). For German examples, see Alexander Stöger, “Constructing the Persona of the Naturwissenschaftler: German Book Reviews on Galvanism,” *Studia Historiae Scientiarum* 20 (2021): 681–709; Christiaan Engberts, “Scholarship, Community Formation and Book Reviews: The *Literarisches Centralblatt* as Arena and Meeting Place,” *Studia Historiae Scientiarum* 20 (2021): 651–79.

72. *PT* papers, quoted in David Kaiser, “The Origins of Physics Today,” *PT* 71 (2018): 35. See also Boring, “CP Speaks,” p.13 (note 71).

73. We studied multiple issues of *PT* to compensate for the fact that fewer book reviews were published in this journal compared to *AHR* and *CP*; this suggests that book reviews played a more prominent role in history and psychology than in physics.

74. E.G. Boring, “Comment to Reviewers,” *CP* 12 (1967): 395–6.

75. Richard Camp, “Review of *The Emergence of Political Catholicism in Italy*, by John N. Molony,” *AHR* 83 (1978): 208; Laird Thompson, “Review of *Adaptive Optics for Astronomical Telescopes*, by John W. Hardy and François Roddier,” *PT* 53 (2000): 69.

76. Jeri E. Doane, “Review of *Towards Need-Specific Treatment of Schizophrenic Psychoses*, by Yrjo O. Alanen et al.,” *CP* 34 (1989): 48–9, 49; Louis D. Roberts, “Review of *Quantum Theory of Solids*, by R.E. Peierls,” *PT* 9 (1956): 29.

77. Robert H. Austin, “Review of *The Touchstone of Life*, by Werner R. Loewenstein,” *PT* 53 (2000): 59–60, 60, 59.

78. Stanley G. Payne, “Review of *Privilege, Persecution, and Prophecy*, by Frances Lannon,” *AHR* 94 (1989): 157–8, 157.

79. In our sample, (variations of) the virtue-qualifier “judicious” occurs twenty-three times in *AHR*, once in *PT*, and not at all in *CP*.

80. Boring, “Comment to Reviewers,” pp. 395–6 (note 74).

81. S. William Harperin, “Review of *Léon Blum*, by Joel Colton,” *AHR* 72 (1967): 604–5, 604.

82. Phillip Zimbardo, “Review of *Shyness*, by Bernardo J. Carducci,” *CP* 45 (2000): 99–101.

83. Helmut Hirsch, “Review of *Freiheitsliebende Rheinlander*, by Andrew Lees,” *AHR* 83 (1978): 189–90, 190.

84. Hirsch Lazaar Silverman, “Review of *Changing Views of the Human Condition*, by Paul W. Pruyser,” *CP* 34 (1989): 41.

85. John D. Fair, “Review of *Ernie O’Malley*, by Richard English,” *AHR* 105 (2000): 288–9, 288.

86. Harold G. McCurdy, “Review of *Freud on Broadway*, by W. David Sievers,” *CP* 1 (1956): 12, 12.

87. Leo M. Hurvich and Dorothea Jameson, “Review of *Vision and Visual Perception*, by Clarence Graham,” *CP* 12 (1967): 3–5, 3; D. W. Brogan, “Review of *Conservatism in America*, by Clinton Rossiter,” *AHR* 61 (1956): 410–12, 411; Elizabeth Douvan, “Review of *Aspects of the Analysis of Family Structure*, by Ansley J. Coale et al.,” *CP* 12 (1967): 13.

88. Mark A. Edinberg, “Review of *Dying*, by Hannelore Wass, Felix M. Berardo, and Robert A. Neimeyer,” *CP* 34 (1989): 75; Faye J. Crosby and Carmel Benson, “Review of *Pragmatic Women and Body Politics*, by Margaret M. Lock and Patricia A. Kaufert,” *CP* 45 (2000): 51–2, 52.

89. Marianne DeKoven, “Review of *The Gender of History*, by Bonnie G. Smith,” *AHR* 105 (2000): 167–8, 168. Objectivity and empathy were applied as virtue-qualifiers as well, for example by a psychologist who noted that “those who find congenial the objective analyses and unemotional reports of experience will be repelled by the imaginative, emotionally charged essays” presented in one book. Jerome D. Frank, “Review of *Heal or Die*, by Kenneth Porter, Deborah Rinzler, and Paul Olsen,” *CP* 34 (1989): 72, 72.

90. There was reference to “objectivity,” however, in the code of ethics published by the APS, “APS Council Adopts Statement on Integrity in Physics,” *PT* 40 (1987): 81. <https://doi.org/10.1063/1.2820092>.

91. T. Teichmann, “Review of *An Introduction to Probability Theory and its Applications*, vol. 2, by William Feller,” *PT* 20 (1967): 76, 76; Freeman J. Dyson, “Review of *Mesons and Fields*, vol. 1, by S. S. Schweber, H. A. Bethe, and F. De Hoffmann,” *PT* 9 (1956): 32–4, 33–4.

92. Noble E. Cunningham, “Review of *The Thinking Revolutionary*, by Ralph Lerner,” *AHR* 94 (1989): 214; Sidney Drell, “Review of *‘What Do You Care What Other People Think?’* by Richard P. Feynman,” *PT* 42 (1989): 106–7; Dyson, “Review of *Mesons and Fields*” (note 91).

93. McCurdy, “Freud on Broadway,” 12 (note 86); Merrill Moore, “Review of *Management of Addictions*, by Edward Podolsky,” *CP* 1 (1956): 23, 23.

94. Karl W. Deutsch, “Review of *Das Nationale als Europäisches Problem* by Reinhard Wittram and *L’idée de la nationalité* by Pierre Vergnaud,” *AHR* 61 (1956): 394–6.

95. Laura Stark, “The Science of Ethics: Deception, the Resilient Self, and the APA Code of Ethics 1966–1973,” *Journal of the History of the Behavioral Sciences* 46 (2010): 337–70, 337; Roger Cooter, “The Resistible Rise of Medical Ethics,” *Social History of Medicine* 8 (1995): 257–70, 262. On the kind of negotiations surrounding the drafting of ethics codes, see Stark’s article and several chapters in Robert B. Baker et al. (eds.), *The American Medical Ethics Revolution: How the AMA’s Code of Ethics Has Transformed Physicians’ Relationships to Patients, Professionals, and Society* (Baltimore, MD: Johns Hopkins University Press, 1999).

96. The 1974 AHA code, for instance, claimed to “reflect opinions and values developed by [the discipline’s scholars] over a long period of time.” AHA, “Statement of Professional Standards” (1974), <https://www.historians.org/about-aha-and-membership/aha-history-and-archives/historical-archives/report-of-the-aha-committee-on-the-rights-of-historians-(1974)/iv-statement-of-professional-standards> (November 11, 2023).

97. APS, “Guidelines for Professional Conduct” (1991) (note 90).

98. Ibid. See also APS, “Guidelines for Professional Conduct” (2002), <https://www.aps.org/policy/statements/02_2.cfm> (November 11, 2023).

99. AHA, “Statement on Standards of Professional Conduct” (2005), <https://chnm.gmu.edu/digitalhistory/links/cached/chapter6/6_23f_professionalstandards.htm> (November 11, 2023).

100. AHA, “Statement of Professional Standards” (1974) (note 96); APA, “Ethical Principles of Psychologists and Code of Conduct,” *American Psychologist* 47, no. 12 (1992): 1597–1611; APA, “Ethical Standards of Psychologists,” *American Psychologist* 14 (1959): 279–82, 279; AHA, “Statement on Standards of Professional Conduct,” *The History Teacher* 21, no. 1 (1987): 105–9, 105, 107; AHA, “Statement on Standards of Professional Conduct” (1999), <https://www.concernedhistorians.org/content_files/file/et/223.pdf> (November 15, 2023).

101. APA, “Ethical Standards of Psychologists” (1959), p. 279 (note 100); APA, “Ethical Principles of Psychologists and Code of Conduct” (1992), p. 1599 (note 100).

102. On debates around classifying integrity as a virtue, see Charlotte Alston, Amber D. Carpenter, and Rachael Wiseman, “Introduction,” in Charlotte Alston, Amber D. Carpenter, and Rachael Wiseman (eds.), *Portraits of Integrity: 26 Case Studies from History, Literature and Philosophy* (London: Bloomsbury, 2020), pp.1–14, 2–3.

103. APA, “Ethical Principles of Psychologists and Code of Conduct” (1992), p. 1599 (note 100).

104. AHA, “Statement of Professional Standards” (1974) (note 96); similarly AHA, “Statement on Standards of Professional Conduct” (1987), p. 107 (note 100) and AHA, “Statement on Standards of Professional Conduct” (1999) (note 100).

105. AHA, “Statement on Standards of Professional Conduct” (1987), p. 105 (note 100). Also AHA, “Statement of Professional Standards” (1974) (note 96), and AHA, “Statement on Standards of Professional Conduct” (1999) (note 100).

106. AHA, “Statement of Professional Standards” (1974) (note 96).

107. See APA, “Ethical Standards of Psychologists” (1959), (note 100); APA, “Ethical Standards of Psychologists,” *American Psychologist*, 36, no. 6 (1981): 633–38; APA, “Ethical Standards of Psychologists,” *APA Monitor* 8, no. 3 (1977): 22–3; APA, “Ethical Principles of Psychologists” (1981), <http://ethicscodescollection.org/detail/7c01cd5e-e6b2-4b88-aaa3-1ab6255ab46c> (July 11, 2023); APA, “Ethical Principles of Psychologists and Code of Conduct” (1992), (note 100). Competence also appears as an important personal quality in the 1953 APA code: “Ethical Standards of Psychologists,” Washington, 1953.

108. This wording remains identical in ethics codes from 1959 to at least 1981, e.g. APA, “Ethical Standards of Psychologists” (1959) (note 100), p. 279.

109. E.g. APA, “Ethical Standards of Psychologists” (1959), (note 100), p. 279.

110 APA, “Ethical Standards of Psychologists” (1953), p.99 (note 107); APA, “Ethical Standards of Psychologists” (1963), p. 56 (note 107); APA, “Ethical Principles of Psychologists” (1981) (note 107).

111. Cohen-Cole, *The Open Mind* (note 20).

112. These last two appear only as virtue-qualifiers, in line with the trend in textbooks and book reviews.

113. See APA, “Ethical Standards of Psychologists” (1963) (note 107); APA, “Ethical Principles of Psychologists” (1981) (note 107). As “public responsibility” in APA, “Ethical Standards of Psychologists” (1953) (note 107), “social responsibility” in APA, “Ethical Principles of Psychologists and Code of Conduct” (1992) (note 100).

114. Serge P. J. M. Horbach and Willem Halffman, “Promoting Virtue or Punishing Fraud: Mapping Contrasts in the Language of ‘Scientific Integrity,’” *Science and Engineering Ethics* 23 (2017): 1461–85, 1461.

115. “Scholars must be not only competent . . . but cognizant of issues of professional conduct. Integrity is one of these issues” (AHA, “Statement on Standards of Professional Conduct” (1987), 105 [note 100]). The two quotations are from APA, “Ethical Standards of Psychologists” (1963), p.56 (note 107).

116. Mita Giacomini et al., “The Policy Analysis of ‘Values Talk’: Lessons from Canadian Health Reform,” *Health Policy* 67 (2004): 15–24; Mita Giacomini, Nuala Kenny, and Deirdre DeJean, “Ethics Frameworks in Canadian Health Policies: Foundation, Scaffolding, or Window Dressing?,” *Health Policy* 89 (2009): 58–71; Horbach and Halffman, “Promoting Virtue” (note 114). Rik Peels and co-authors similarly find “value pluralism” in contemporary codes of research integrity. Rik Peels et al., “Value Pluralism in Research Integrity,” *Research Integrity and Peer Review* 4 (2019): 18.

117. Giacomini et al., “Policy Analysis,” p. 22 (note 116) find “values talk” in health policy documents to be “paradoxically, both very important and ambiguous in its meaning.”

118. Cf. Giacomini et al., “Policy Analysis,” p. 21 (note 116).

119. APS, “Guidelines for Professional Conduct” (1991) (note 90); APS, “Guidelines for Professional Conduct” (2002) (note 98).

120. AHA, “Statement on Standards of Professional Conduct” (2005) (note 99).

121. APA, “Ethical Standards of Psychologists” (1953), p.94 (note 107).

122. APA, “Ethical Principles of Psychologists and Code of Conduct” (1992), p.1607 (note 100).

123. AHA, “Statement on Standards of Professional Conduct” (1987), p.108 (note 100). Similar wording in AHA, “Statement of Professional Standards” (1974) (note 96).

124. APA, “Ethical Standards of Psychologists” (1959), p.280 (note 100); APA, “Ethical Standards of Psychologists” (1963), p.57 (note 107).

125. AHA, “Statement on Standards of Professional Conduct” (1987), p.106 (note 100).

126. APA, “Ethical Principles of Psychologists” (1981) (note 107).

127. AHA, “Statement of Professional Standards” (1974) (note 96).

128. Their regulatory nature was already implicit in the term “standards,” with its normative implications, which headed the AHA and APA documents.

129. APA, “Ethical Principles of Psychologists and Code of Conduct” (1992), p.1598 (emphasis in original) (note 100).

130. Ibid., p.1598.

131. APA, “Ethical Standards of Psychologists” (1953), p.142 (note 107).

132. Larry Laudan, “More on Creationism,” *Science, Technology, and Human Values* 8 (1983): 36–8, 37.

133. AHA, “Statement on Standards of Professional Conduct” (2005) (note 99).

134. Hilgard, *Introduction to Psychology*, 3rd ed., p.563 (note 40); Ruch and Zimbardo, *Psychology and Life*, 8th ed., pp.14–15 (note 50); Ruch, *Psychology and Life*, 7th ed., p.138 (note 52); Gleitman, *Psychology*, 2nd ed., p.429 (note 63). See also Myers, *Psychology*, 2nd ed., p.319 (note 54) and David G. Myers, *Psychology*, 5th ed. (New York: Worth, 1998), p.36.

135. Garraghan, *A Guide to Historical Method*, pp.46–53 (note 37); Shafer, *Guide to Historical Method*, pp.169–74 (note 61).

136. Jacques Barzun and Henry A. Graff, *The Modern Researcher* (New York: Harcourt, Brace, Jovanovich, 1957), p.163.

137. Ibid., p.153. Norman F. Cantor and Richard I. Schneider, *How to Study History* (Arlington Heights, IL: AHM Publishing, 1967), p.20, went even further by arguing that the historian’s subjectivity should be welcomed as a potential source of insight rather than an obstacle.

138. AHA, “Statement on Standards of Professional Conduct” (1987) (note 100).

139. Paul, *Historians’ Virtues*, p.47 (note 5) observes a shift in emphasis from “virtues of restraint” to “virtues of transparency.” Our finding also accords with Daston and Galison’s account of a move from the ideal of “mechanical objectivity,” and an associated self-effacement, to a regime of “trained judgment.” Daston and Galison, *Objectivity* (note 17).

140. Horbach and Halffman, “Promoting Virtue,” p.1481 (note 114); Lissa L. Roberts, H. Otto Sibum, and Cyrus C. M. Mody, “Integrating the History of Science into Broader Discussions of Research Integrity and Fraud,” *History of Science* 58 (2020): 354–68, 360.

141. Cf. Horbach and Halffman, “Promoting Virtue,” pp.1464–6 (note 114). We find some indications that talk about integrity increased around the last decade of the century. In 1992, integrity appeared as the heading of a principle in the APA code, while the APS couched its 1987 dismissal of the need for any “formal code of ethics” by referring to physicists’ “well-deserved reputation for maintenance of high ethical standards and integrity.”

142. Shapin, *The Scientific Life*, p.13 (note 10).

143. Kathryn M. Olesko, *Physics as a Calling: Discipline and Practice in the Königsburg Seminar for Physics* (Ithaca, NY: Cornell University Press, 1991), pp.366–450.

144. Rutherford, “Maintaining Masculinity” (note 23); Chanda Prescod-Weinstein, “Making Black Women Scientists under White Empiricism: The Racialization of Epistemology in Physics,” *Signs: Journal of Women in Culture and Society* 45 (2020): 421–47. On “scientific self-sacrifice” in an earlier period, see Rebecca Herzig, *Suffering for Science: Reason and Sacrifice in Modern America* (Piscataway, NJ: Rutgers University Press, 2005).

145. Robert Baker, *Before Bioethics: A History of American Medical Ethics from the Colonial Period to the Bioethics Revolution* (Oxford: Oxford University Press, 2013); Thomas H. Bivins, “The Language of Virtue: What Can We Learn from Early Journalism Codes of Ethics?,” in Wendy N. Wyatt (ed.), *The Ethics of Journalism: Individual, Institutional and Cultural Influences* (London: I. B. Tauris, 2014), pp.165–84.

146. Samuel Schindler, *Theoretical Virtues in Science: Uncovering Reality through Theory* (Cambridge: Cambridge University Press, 2018). On “epistemic virtues” as a historiographical category, see van Dongen and Paul, “Introduction” (note 14).

## Appendix: List of sources

**Table A1.** Corpus of textbooks.

*Physics textbooks* 1. Francis Weston Sears and Mark W. Zemansky, *University Physics*, 2nd ed. (Cambridge, MA: Addison-Wesley, 1955). 2. Robert Resnick and David Halliday, *Physics for Students of Science and Engineering*, 2 vols, 2nd ed. (New York: John Wiley & Son, 1962). 3. Richard P. Feynman, Robert B. Leighton, and Matthew Sands, *The Feynman Lectures on Physics*, vol. 1. (Redwood City, CA: Addison-Welsey, 1963). 4. Marcelo Alonso and Edward J. Finn, *Fundamental University Physics*, vol. 1, 1st ed. (Reading, MA: Addison-Wesley, 1967). 5. Daniel Kleppner and Robert J. Kolenkow, *An Introduction to Mechanics*, 1st ed. (New York: McGraw-Hill, 1973). 6. Francis Weston Sears, Mark W. Zemansky, and Hugh D. Young, *University Physics*, 5th ed. (Reading, MA: Addison-Wesley, 1976). 7. Paul A. Tipler, *Physics*, 1st ed. (New York: Worth, 1976). 8. Robert Resnick and David Halliday, *Physics*, 2 vols., 3rd ed. (New York: John Wiley & Son, 1977). 9. Kenneth S. Krane, *Modern Physics*, 1st ed. (New York: John Wiley & Sons, 1983). 10. Francis Weston Sears, Mark W. Zemansky, and Hugh D. Young, *University Physics*, 7th ed. (Reading, MA: Addison-Wesley, 1987). 11. Daryl W. Preston and Eric R. Dietz, *The Art of Experimental Physics*, 1st ed. (New York: John Wiley & Sons, 1991). 12. Robert Resnick, David Halliday, and Kenneth S. Krane, *Physics*, 2 vols., 4th ed. (New York: John Wiley & Sons, 1992). *Psychology textbooks* 13. Norman L. Munn, *Psychology: The Fundamentals of Human Adjustment* (Boston, MA: Houghton Mifflin, 1946). 14. Ernest R. Hilgard, *Introduction to Psychology*, 1st ed. (New York: Harcourt, Brace, and Company, 1953). 15. Clifford T. Morgan and Richard Austin King, *Introduction to Psychology*, 1st ed. (New York: McGraw-Hill, 1956). 16. Floyd L. Ruch, *Psychology and Life*, 5th ed. (Chicago, IL: Scott, Foresman, 1958). 17. Ernest R. Hilgard, *Introduction to Psychology*, 3rd ed. (New York: Harcourt, Brace, and World, 1962). 18. Clifford T. Morgan and Richard Austin King, *Introduction to Psychology*, 3rd ed. (New York: McGraw-Hill, 1966). 19. Floyd L. Ruch, *Psychology and Life*, 7th ed. (Chicago, IL: Scott, Foresman, 1967). 20. Floyd L. Ruch and Philip G. Zimbardo, *Psychology and Life*, 8th ed. (Glenview, IL: Scott, Foresman, 1971). 21. Ernest R. Hilgard, Richard C. Atkinson, and Rita L. Atkinson, *Introduction to Psychology*, 5th ed. (New York: Harcourt, Brace, and World, 1971). 22. Clifford T. Morgan and Richard Austin King, *Introduction to Psychology*, 5th ed. (New York: McGraw-Hill, 1975). 23. Rita L. Atkinson, Richard C. Atkinson, and Ernest R. Hilgard, *Introduction to Psychology*, 8th ed. (San Diego, CA: Harcourt, Brace, Jovanovich, 1983). 24. Henry Gleitman, *Psychology*, 2nd ed. (New York: W. W. Norton, 1986). 25. David G. Myers, *Psychology*, 2nd ed. (New York: Worth, 1989). 26. Clifford T. Morgan et al., *Introduction to Psychology*, 7th ed. (New Delhi: McGraw-Hill Education, 1993). 27. Henry Gleitman, *Psychology*, 4th ed. (New York: W. W. Norton, 1995). 28. Rita L. Atkinson et al., *Introduction to Psychology*, 12th ed. (Orlando, FL: Harcourt Brace, 1996). 29. David G. Myers, *Psychology*, 5th ed. (New York: Worth, 1998).
*History textbooks* 30. Gilbert J. Garraghan, *A Guide to Historical Method*, Jean Delanglez (ed.) (New York: Fordham University Press, 1946). 31. Jacques Barzun and Henry A. Graff, *The Modern Researcher* (New York: Harcourt, Brace, Javanovich, 1957). 32. Norman F. Cantor and Richard I. Schneider, *How to Study History* (Arlington Heights, IL: AHM Publishing, 1967). 33. Louis Gottschalk, *Understanding History: A Primer of Historical Method*, 2nd ed. (New York: Alfred A. Knopf, 1969). 34. Robert Jones Shafer (ed.), *A Guide to Historical Method*, 2nd ed. (Homewood, IL: Dorsey Press, 1974). 35. Jacques Barzun and Henry A. Graff, *The Modern Researcher*, 4th ed. (New York: Harcourt, Brace, Jovanovich, 1977). 36. Robert V. Daniels, *Studying History: How and Why*, 3rd ed. (Englewood Cliffs, NJ: Prentice-Hall, 1981). 37. Jacques Barzun and Henry A. Graff, *The Modern Researcher*, 5th ed. (New York: Harcourt, Brace, Javanovich, 1985). 38. Conal Furay and Michael J. Salevouris, *The Methods and Skills of History: A Practical Guide* (Arlington Heights, IL: H. Davidson, 1988).

**Table A2.** Society codes of ethics.

*American Physical Society (APS)* 1. “APS Council Adopts Statement on Integrity in Physics.” *Physics Today* 40 (1987): 81. https://doi.org/10.1063/1.2820092 2. “Guidelines for Professional Conduct” (1991), <https://www.aps.org/policy/statements/91_8.cfm> (November 15, 2023). 3. “Guidelines for Professional Conduct” (2002), <https://www.aps.org/policy/statements/02_2.cfm> (November 15, 2023). *American Historical Association (AHA)* 4. “Statement of Professional Standards” (1974), <https://www.historians.org/about-aha-and-membership/aha-history-and-archives/historical-archives/report-of-the-aha-committee-on-the-rights-of-historians-(1974)/iv-statement-of-professional-standards> (November 15, 2023). 5. “Statement on Standards of Professional Conduct,” *The History Teacher* 21, no. 1 (1987): 105–9. 6. “Statement on Standards of Professional Conduct” (1999), <https://www.concernedhistorians.org/content_files/file/et/223.pdf> (November 15, 2023). 7. “Statement on Standards of Professional Conduct” (2005), <https://chnm.gmu.edu/digitalhistory/links/cached/chapter6/6_23f_professionalstandards.htm> (November 15, 2023). *American Psychological Association (APA)* 8. “Ethical Standards of Psychologists,” Washington, 1953. 9. “Ethical Standards of Psychologists,” *American Psychologist* 14 (1959): 279–82. 10. “Ethical Standards of Psychologists,” *American Psychologist* 18, no. 1 (1963): 56–60. 11. “Ethical Standards of Psychologists,” *American Psychologist* 23, no. 5 (1968): 357–61. 12. “Ethical Standards of Psychologists,” *APA Monitor* 8, no. 3 (1977): 22–3. 13. “Ethical Principles of Psychologists,” *American Psychologist*, 36, no.6 (1981): 633–38. 14. “Ethical Principles of Psychologists and Code of Conduct,” *American Psychologist* 47, no. 12 (1992): 1597–1611.

